# Supplementary material for: Structure of Leishmania donovani 6-Phosphogluconate Dehydrogenase and Inhibition by Phosphine Gold(I) Complexes: A Potential Approach to Leishmaniasis Treatment
Source: Int J Mol Sci. 2023 May 11;24(10):8615. doi: 10.3390/ijms24108615 (PMC10217883; doi:10.3390/ijms24108615)
Supplement: Supplementary file 1 [file ijms-24-08615-s001.zip › ijms-2340972-supplementary.pdf]

Supplementary Material

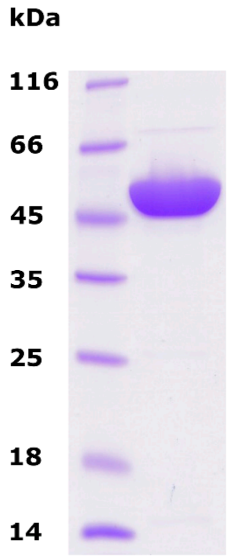

Figure S1. Coomassie-stained SDS-PAGE gel (12% polyacrylamide) of purified *Ld*6PGD wt. N-terminally His-tagged *Ld*6PGD was purified via Ni-NTA affinity chromatography followed by size exclusion chromatography (SEC). Left: unstained protein molecular weight marker (Thermo Scientific, Dreieich, Germany). Molecular weight of *Ld*6PGD wt: 55.4 kDa.

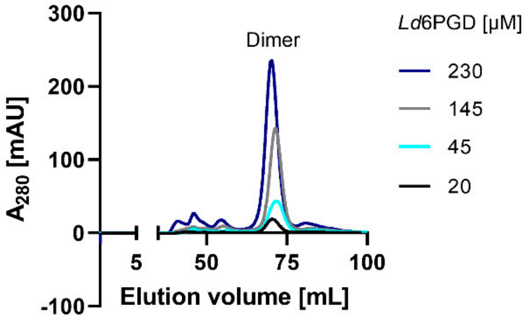

Figure S2. SEC analysis of recombinant *Ld*6PGD wt under different enzyme concentrations. 230 µM (purple, rv = 70.2 mL ≙ 111 kDa), 145 µM (grey, rv = 71.5 mL ≙ 99.9 kDa), 45 µM (cyan, rv = 71.8 mL ≙ 97.5 kDa) and 20 µM (black, rv = 70.6 mL ≙ 107.5 kDa) revealed identical elution patterns with one peak equivalent to a dimer. Representative chromatograms (n ≥ 2) are shown for each condition.

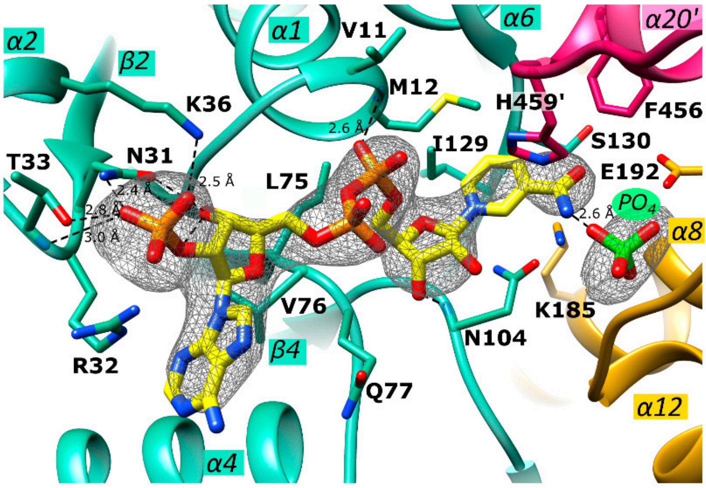

Figure S3. Active site close-up of *Ld*6PGD with omit map. The active site of monomer A is shown. Ribbons and residues of domain 1 are colored light green and of domain 2 gold. The C-terminal part of domain 2 of monomer B is colored magenta. The NADP(H) moiety is colored yellow. Within the 6PG binding pocket a phosphate ion (PO_4) is visible (green). Electron density map (F_O-F_C omit map, calculated without NADP(H) and PO_4) contoured at 3.0 σ for NADP(H) and PO_4 is shown in black. Residues of the $NADP^+$ and the 6PG binding pocket are shown in stick models, and hydrogen bonds are indicated with black dotted lines.
